# Letter to the Editor: Mean Platelet Volume to Platelet Count Value May Not Be a Prognostic Marker in Patients with Crimean-Congo Hemorrhagic Fever

**DOI:** 10.5041/RMMJ.10420

**Published:** 2020-10-14

**Authors:** Cengiz Beyan, Esin Beyan

**Affiliations:** 1 Retired Professor in Hematology, Ufuk University Faculty of Medicine, Ankara, Turkey; 2 Professor, University of Health Sciences, Kecioren Training and Research Hospital, Department of Internal Medicine, Ankara, Turkey

## TO THE EDITOR

We read with great interest the retrospective article of Tekin and Engin that investigated the prognostic significance of the ratio of mean platelet volume (MPV) to platelet count ratio (MPVPCR) in patients with Crimean-Congo hemorrhagic fever (CCHF).^1^ The authors found that MPVPCR was significantly lower in survivors than in non-survivors, and therefore they suggested that this ratio could be used as a mortality marker. We think there are other factors that might have affected the results of this study.

First of all, the fact that the study was carried out in a retrospective rather than prospective nature prevented the elimination of pre-analytical and analytical errors. Also, the fact that the data belong to a very wide period of time, namely eight-and-a-half years, raises the concern that the methods and devices used in the measurement may have changed. Moreover, both of the compared groups were diseased (i.e. survivors and non-survivors), and the absence of a healthy control group made it difficult to understand the meaning of the results obtained.

At the time of writing, standardization of MPV measurement has not been achieved.^2^–^4^ The measurement results of MPV can vary considerably depending on reasons such as the time from venipuncture to measurement, the usage of ethylenediaminetetraacetic acid (EDTA) or citrate as an anti-coagulant, and/or which device is used. When EDTA anticoagulant is used, the ratio can change by 2%–50% depending on the time from venipuncture to measurement.^2^,^5^ Several authors have found that different anticoagulants and/or devices used in MPV measurements also lead to significant deviations in the results.^2^,^3^,^5^–^7^ Since there is no standardization in MPV measurement, Noris et al. have stated that MPV measurement cannot be used as a diagnostic or prognostic marker in acquired diseases.^4^ Because the anticoagulants used in the complete blood count, the time until the measurement after venipuncture, and the devices used in the measurement in Tekin and Engin’s study were not defined, it is highly controversial that the results were deemed reliable. Additionally, although a cut-off value has been specified by Tekin and Engin for MPVPCR levels, it is not possible to define a universal cut-off value as different devices used in the measurement give different results.^6^,^7^

In conclusion, MPVPCR value may not be a prognostic marker in patients with CCHF.

## Abbreviations

CCHF: Crimean-Congo Hemorrhagic Fever
EDTA: ethylenediaminetetraacetic acid
MPV: mean platelet volume
MPVPCR: MPV to platelet count ratio
